# Loss of ARID1A expression sensitizes cancer cells to PI3K- and AKT-inhibition

**DOI:** 10.18632/oncotarget.2092

**Published:** 2014-06-11

**Authors:** Eleftherios P Samartzis, Katrin Gutsche, Konstantin J Dedes, Daniel Fink, Manuel Stucki, Patrick Imesch

**Affiliations:** ^1^ Department of Gynecology, University Hospital of Zurich, Zürich, Switzerland

**Keywords:** ARID1A, BAF250a, SWI/SNF, PI3K/AKT pathway, AKT phosphorylation, PIK3CA, apoptosis, AKT-inhibitor, MK-2206, perifosine, PI3K-inhibitor, buparlisib (BKM120), ovarian clear cell carcinomas, endometriosis associated ovarian carcinomas, endometrial cancer, breast cancer

## Abstract

*ARID1A* mutations are observed in various tumors, including ovarian clear cell (OCCC) and endometrioid carcinomas, endometrial, and breast carcinomas. They commonly result in loss of ARID1A-protein expression and frequently co-occur with PI3K/AKT-pathway activating mechanisms. The aim of this study was to test the hypothesis as to whether PI3K/AKT-pathway activation is a critical mechanism in *ARID1A*-mutated tumors and if consequently ARID1A-deficient tumors show increased sensitivity to treatment with PI3K- and AKT-inhibitors. Upon *ARID1A* knockdown, MCF7 breast cancer cells and primary MRC5 cells exhibited a significantly increased sensitivity towards the AKT-inhibitors MK-2206 and perifosine, as well as the PI3K-inhibitor buparlisib. Knockdown of *ARID1A* in MCF7 led to an increase of pAKT-Ser^473^. AKT-inhibition with MK-2206 led to increased apoptosis and to a decrease of pS6K in ARID1A-depleted MCF7 cells but not in the controls. In five OCCC cell lines ARID1A-deficiency correlated with increased pAKT-Ser^473^ levels and with sensitivity towards treatment with the AKT-inhibitor MK-2206. In conclusion, ARID1A-deficient cancer cells demonstrate an increased sensitivity to treatment with small molecule inhibitors of the PI3K/AKT-pathway. These findings suggest a specific requirement of the PI3K/AKT pathway in ARID1A-deficient tumors and reveal a synthetic lethal interaction between loss of ARID1A expression and inhibition of the PI3K/AKT pathway.

## INTRODUCTION

Mutations in the gene encoding the *AT-rich interacting domain containing protein 1A (ARID1A)* are frequently observed in a wide variety of gynecological and non-gynecological cancers [1, 2]. These occur in approximately 50% of endometriosis-associated ovarian clear cell (OCCC) and 30% of endometrioid ovarian carcinomas (EnOC) [3, 4], in endometrial carcinomas, with a loss of expression in 20-30% depending on the histological subtype [5, 6], as well as in breast carcinomas (mutations in 4-35%) [7, 8]. Non-gynecological carcinomas with frequent ARID1A mutations include pancreatic carcinomas (mutations in 8-45%) [9, 10], gastric adenocarcinomas (mutations in 8-29%) [11-13], hepatocellular carcinomas (mutations in 10-17%) [14-16], as well as clear cell renal cell carcinomas [17, 18]. The majority of the mutations lead to a loss of the ARID1A encoded protein [3], also referred to as BAF250a or p270, which is a subunit of the SWI/SNF chromatin remodeling complex [2]. Although *ARID1A* has recently been identified as a *bona fide* tumor suppressor gene and is currently being intensively investigated, the knowledge about the function and the consequences of a loss of expression of this protein is relatively limited [2].

Interestingly, *ARID1A* mutations frequently coexist with activating mutations of *PIK3CA* [12, 19] and/or loss of PTEN expression [20], which both lead to a downstream activation of the PI3K/AKT pathway. Furthermore, it has recently been shown in endometrial cancer that loss of ARID1A expression leads to an increased phosphorylation of AKT at Ser-473[21]. Similarly, increased AKT phosphorylation has also been reported in OCCC tissue samples with loss of ARID1A expression when concomitant *PIK3CA* mutations and loss of PTEN expression were excluded [22].

These observations strongly suggest interdependency between *ARID1A* mutations and PI3K/AKT pathway activation, indicating that tumor cells with loss of ARID1A expression may be dependent on constitutive activation of the PI3K/AKT-pathway and consequently may also be more vulnerable to its inhibition [23]. This is of considerable clinical relevance since loss of ARID1A expression may be predictive for a favorable treatment response to small molecule inhibitors of the PI3K/AKT-pathway, which are currently under clinical investigation.

In this study, we demonstrate that depletion of ARID1A protein expression significantly increases the sensitivity of cancer cells towards PI3K- and AKT-inhibitors, which is reflected by increased rates of apoptosis in treated ARID1A-depleted cells. Our findings suggest a dependency of *ARID1A*-mutated tumors on activating mechanisms of the PI3K/AKT-pathway, which may be exploited therapeutically.

## RESULTS

### ARID1A-deficient cell lines demonstrate increased sensitivity to treatment with the AKT-inhibitors MK-2206 and perifosine

**Figure 1:**
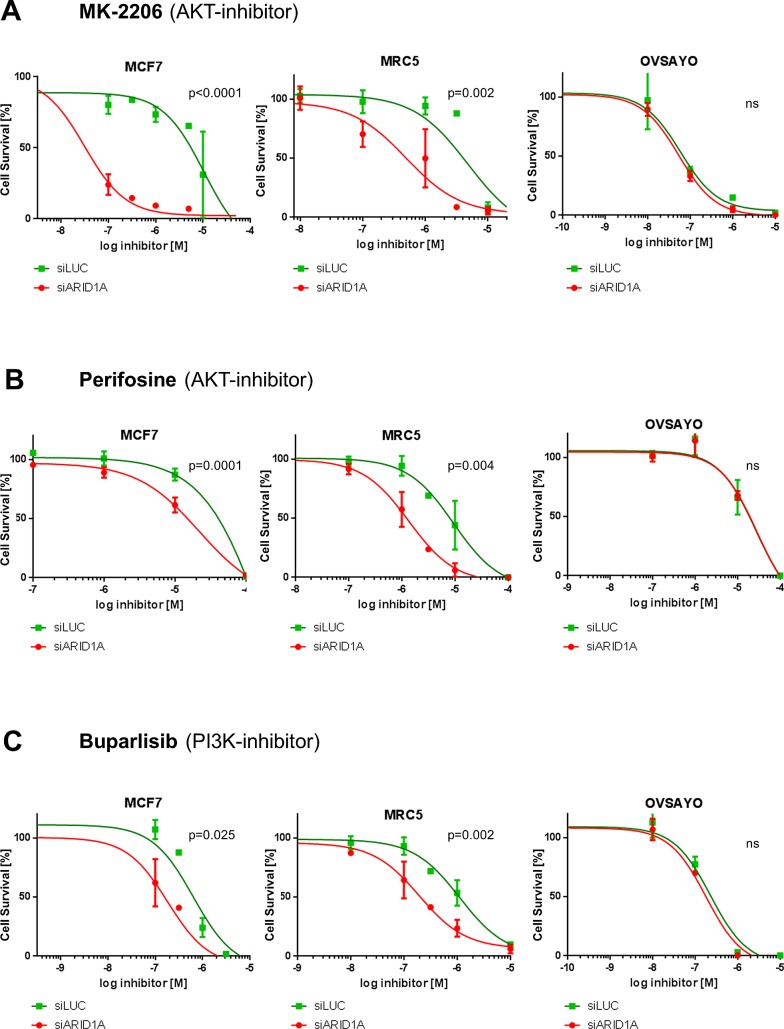
Loss of ARID1A expression leads to increased sensitivity towards the AKT-inhibitors MK-2206 and perifosine as well as towards the PI3K-inhibitor buparlisib in MCF7 and MRC5 cells (A) Increased sensitivity of ARID1A-depleted MCF7 and MRC5 cells towards the AKT-inhibitor MK-2206. (B) Increased sensitivity of ARID1A-depleted MCF7 and MRC5 cells towards the AKT-inhibitor perifosine. (C) Increased sensitivity of ARID1A-depleted MCF7 and MRC5 cells towards the PI3K-inhibitor buparlisib. The ARID1A-deficient OVSAYO cell line served as a control in all the experiments. The p-values indicate the divergence of the IC50-values calculated by an F-test. Significance was assumed for p<0.05 and is indicated in the figures (ns: not significant).

ARID1A-depleted MCF7 cells were found to be significantly more sensitive to treatment with the AKT-inhibitors MK-2206 and perifosine, as demonstrated in Figure 1a and 1b. The LogIC50 values for treatment with MK-2206 were −7.411 ±0.211 in ARID1A-depleted MCF7 cells, when compared to −4.841 ±0.273 in the controls (p<0.0001). This represents a more than 370-fold increased AKT inhibitor sensitivity of ARID1A-depleted MCF7 cells as compared to the wild-type control. LogIC50 for perifosine in ARID1A-depleted MCF7 cells was −4.984 ±0.133, compared to the LogIC50 of −3.774 ±0.423 (p=0.0001) for the controls, which means an approximately 16-fold increased sensitivity.

We performed the same experiment in the primary fibroblast cell line MRC5 to verify our observations in a cell line without any oncogenic mutations and, similarly, we found an increased sensitivity of ARID1A-depleted MRC5 cells towards treatment with MK-2206 with LogIC50 of −6.307 ±0.173 as compared to −5.316 ±0.236 for the control (p=0.002). This meant an approximately 10-fold increase in sensitivity. For treatment with perifosine the LogIC50 values were −5.867 ±0.135 in ARID1A-depleted MRC5 cells, as compared to −5.024 ±0.199 in the controls: equivalent to an increase in sensitivity of approximately 7-fold in ARID1A-depleted MRC5 cells (p=0.004).

As a control, no significant difference in sensitivity to treatment with MK-2206 or perifosine was found in the ARID1A-deficient OCCC cell line OVSAYO after transfection with ARID1A siRNA.

### ARID1A-deficient cell lines demonstrate increased sensitivity to treatment with the PI3K-inhibitor buparlisib

The sensitivity to treatment with the PI3K inhibitor buparlisib in ARID1A-depleted MCF7 and MRC5 cells was shown to increase significantly with comparison to the controls, but less pronounced than for inhibition of AKT (Figure 1c). The LogIC50 values were −6.780 ±0.150 for ARID1A-depleted MCF7 cells, as compared to −6.252 ±0.184 for the controls (approximately a 3-fold increase; p=0.025) and −6.712 ±0.154 for ARID1A-depleted MRC5 as compared to −5.950 ±0.128 for the controls (p=0.002), representing an almost 6-fold increase in sensitivity. The ARID1A-deficient control cell line, OVSAYO, did not show an increased sensitivity for buparlisib upon transfection with ARID1A siRNA.

### Loss of ARID1A expression increases AKT phosphorylation at Ser-473 and downstream phosphorylation of p70 S6 kinase in MCF7 and MRC5 cells

**Figure 2:**
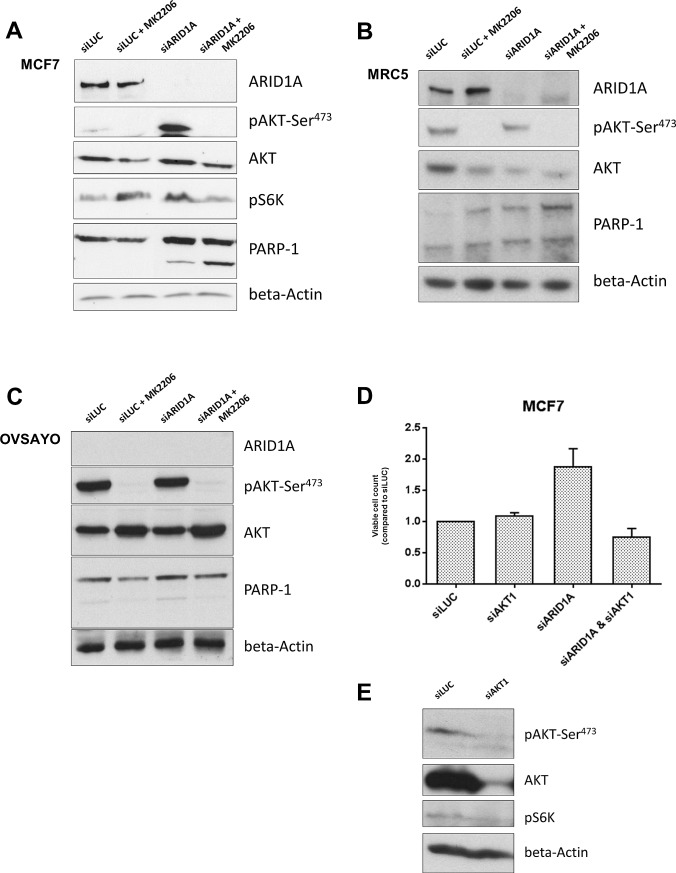
Loss of ARID1A expression increases vulnerability of cancer and primary cells to AKT-inhibition resulting in increased apoptosis (A) Immunoblot showing an increased phosphorylation of AKT at Ser-473 in response to knockdown of *ARID1A* by siRNAs in the MCF7 cell line. ARID1A depletion increased pS6K downstream. Treatment with the AKT-inhibitor MK-2206 (at a concentration of 10^−6^M) completely abrogated pAKT-Ser^473^ in ARID1A-deficient MCF7 cells and led to reduced pS6K, in contrast to the controls where pS6K was not reduced. PARP-1 cleavage was markedly increased in ARID1A-deficient MCF7 cells treated with MK-2206 indicating an increased apoptosis rate, in contrast to the controls where no increase of the apoptosis rate was detectable after treatment with MK-2206. (B) Immunoblot demonstrating *ARID1A* knockdown in MRC5 cell line. The relative level of pAKT-Ser^473^ compared to the respective AKT level was increased in ARID1A-depleted MRC5 cells and completely abrogated by the treatment with the AKT-inhibitor MK-2206 (10^−6^M). (C) Immunoblot demonstrating the effects of a treatment with the AKT-inhibitor MK-2206 (10^−6^M) in the ARID1A-deficient OCCC cell line OVSAYO, which was used as a negative control for the knockdown experiments. Knockdown of *ARID1A* by siRNAs did not show an effect on pAKT-Ser^473^ and PARP-1 cleavage in this cell line, confirming that the effects are specifically due to the knockdown of the *ARID1A* gene. Combination of *ARID1A*-knockdown and treatment with MK-2206 did not cause increased PARP-1 cleavage in this control cell line. (D) MCF7 cells were seeded in 96-well plates and transfected with siLUC, siAKT1, siARID1A, or double-transfected with siAKT1 and siARID1A. Viable cells were counted with an MTS assay after 5 days (indicated as percent of viable cells in relation to the siLUC-transfected controls). *ARID1A* knockdown led to an increased proliferation of MCF7 cells in comparison to the controls. Knockdown of only AKT1 did reduce measurable pAKT-Ser^473^- and AKT- levels and led to a decreased level of pS6K (as shown in (E)), but did not lead to a difference in the amount of viable MCF7 cells. Combined knockdown with AKT1 in contrast completely abrogated the increased proliferation in ARID1A-depleted MCF7 cells. (E) Western blot showing the decreased expression of pAKT-Ser^473^, AKT, and pS6K 120h after AKT1-siRNA knockdown in MCF7 cells.

First, we verified that loss of ARID1A increases phosphorylation of AKT at Ser-473 (pAKT-Ser^473^) as has been described in endometrial cancer cell lines [21]. In both MCF7 and MRC5 cell lines, ARID1A knockdown by siRNA led to an increased phosphorylation of pAKT-Ser^473^ as well as an increased downstream phosphorylation of p70S6kinase (pS6K) as shown in Figure 2a and 2b.

### AKT-inhibition leads to apoptosis in ARID1A-deficient cells

**Figure 3:**
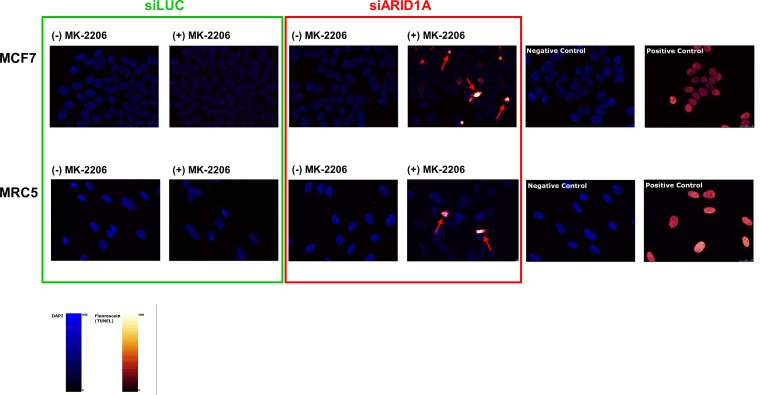
Treatment with the AKT-inhibitor MK-2206 causes apoptosis in ARID1A-depleted MCF7 and MRC5 cells Treatment with MK-2206 (10^−6^M) for 48h led to an increased rate of apoptotic cells in ARID1A-depleted MCF7 and MRC5 cells compared to the controls. Note the different staining pattern of apoptotic cells compared to the positive controls, which is caused by pyknosis and/or fragmentation of the nuclei in the apoptotic cells (indicated by red arrows). The more homogeneous staining in the positive controls is the result of treatment of intact nuclei of fixated non-apoptotic cells with DNase immediately prior to the addition of the TUNEL incubation mix. Negative controls were obtained by omission of the TUNEL enzyme in the incubation mix. The nuclei are represented in blue (DAPI staining) and the fluorescein signal is labelled in red-glow.

We next investigated if inhibition of AKT leads to increased apoptosis in ARID1A-deficient cells. No cleavage of PARP1 (a marker of apoptosis) was observed in the MCF7 control cells (transfected with siLUC) with or without treatment with the AKT-inhibitor, MK-2206. In contrast, PARP1 cleavage was discretely detectable in ARID1A-depleted MCF7 cells and substantially increased after treatment with the AKT-inhibitor MK-2206 (Figure 2a). Similar observations were made for MRC5 (Figure 2b). Consistently, using a TUNEL-assay technique we found that apoptosis was markedly increased in ARID1A-depleted MCF7 and MRC5 cells treated with MK-2206, in comparison to the control cells that expressed normal levels of ARID1A (Figure 3). Upon siARID1A transfection and treatment with MK-2206, PARP1 cleavage was not increased in the ARID1A-deficient control cell line, OVSAYO, which confirmed that the increased apoptosis rate in response to the AKT-inhibitor treatment was specifically due to the knockdown of the *ARID1A* gene and not to other unspecific factors that would be indirectly related to the transfection method (Figure 2c).

### Knockdown of AKT abrogates the increased proliferation rate of ARID1A-depleted MCF7 cells

ARID1A depletion led to an increased proliferation of MCF7 cells in comparison to the controls (Figure 2d). Knockdown of only AKT1 reduced measurable pAKT-Ser^473^- and AKT- levels, and led to a decreased level of pS6K, but did not lead to a difference in the amount of viable MCF7 cells after 5 days (Figure 2d, 2e). In contrast, combined knockdown of ARID1A and AKT1 completely abrogated the increased proliferation in ARID1A-depleted MCF7 cells (Figure 2d).

### Sensitivity to treatment with MK-2206 in OCCC

**Figure 4:**
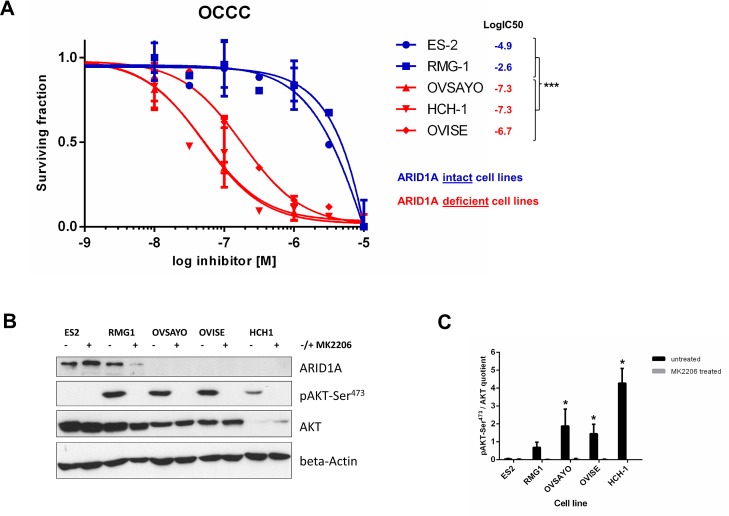
Loss of ARID1A expression is associated with high sensitivity to the AKT-inhibitor MK-2206 in ovarian clear cell carcinoma cell lines (A) The three ARID1A-deficient OCCC cell lines OVSAYO, OVISE, and HCH-1 were highly sensitive to a treatment with MK-2206 whereas the two OCCC cell lines ES-2 and RMG-1 with intact ARID1A expression were resistant to the same treatment. The logIC50 values significantly differed between ARID1A-intact and ARID1A-deficient OCCC cell lines (***p<0.0001) as verified with an F-test (Graph Pad Prism, version 6). (B) Immunoblot showing intact ARID1A expression in ES-2 and RMG-1 and loss of ARID1A expression in OVSAYO, OVISE, and HCH-1. Treatment with MK-2206 (10^−6^M) completely abrogated detectable pAKT-Ser^473^ levels in all OCCC cell lines. (C) Densitometric quantification of the relative pAKT-Ser^473^/AKT expression in the five untreated and treated OCCC cell lines (ImageJ version 1.46, NIH, USA). Relative pAKT-Ser^473^/AKT levels were significantly increased in the three ARID1A-deficient OCCC cell lines OVSAYO, OVISE, and HCH-1, as compared to the two ARID1A-intact OCCC cell lines ES-2 and RMG-1 (*p<0.01) as verified by a t-test (Graph Pad Prism, version 6).

Loss of ARID1A expression correlated with increased pAKT-Ser^473^ in five OCCC cell lines (Figure 4b, 4c). The cell lines, OVSAYO, OVISE, and HCH-1, did not express detectable levels of ARID1A and showed high sensitivity towards treatment with the AKT-inhibitor, MK-2206, whereas the two OCCC cell lines with intact ARID1A expression were resistant towards the same treatment (Figure 4a).

## DISCUSSION

The results of this study demonstrate an interdependency of ARID1A and the PI3K/AKT pathway, which results in significantly increased sensitivity of ARID1A-deficient cancer cells to PI3K- and AKT- inhibition. We thus demonstrate for the first time a principle of synthetic lethality between ARID1A deficiency and inhibition of the PI3K/AKT pathway in two cellular model systems. MCF7 is a well-established breast carcinoma cell line and MRC5 a commonly used human primary lung fibroblast cell line. Although MCF7 contains a moderately activating mutation of PIK3CA [24, 25], depletion of ARID1A by siRNA considerably increased phosphorylation of AKT at Ser-473 in this cell line, as described previously in endometrial carcinoma cell lines [21]. The normal primary fibroblast cell line, MRC5, that does not contain any known mutations [26] confirmed our results, thus minimizing the likelihood of secondary effects by other mutations in the ARID1A knockdown experiments.

Mutations in the *ARID1A* gene are a frequent event occurring in a wide variety of gynecological and non-gynecological cancers [23]. They usually lead to a loss of expression of the *ARID1A* encoded protein [3], and are thus not directly targetable [2]. Indirect therapeutic approaches using the principle of synthetic lethality in regard to members of the SWI/SNF chromatin remodeling complexes are an appropriate alternative strategy [27, 28]. Thus, a recent study demonstrated a synthetic lethal relationship between inactivating mutations of the homologs *ARID1A* and *ARID1B*, both in primary and cancer cells. ARID1B depletion in the context of loss of ARID1A expression led to a significant decrease in cell proliferation revealing this homologue as a relative vulnerability in *ARID1A*-mutant cell lines. Despite the substantial relevance of these observations for potential future approaches, the immediate clinical relevance is limited due to the fact that there are currently no substances available that would therapeutically inhibit ARID1B expression [28].

An interdependency between *ARID1A* mutations and *PIK3CA* is strongly supported by the supplementary data of the study of Helming et al., since *PIK3CA* (just after *ARID1B*) scored as the second top candidate preferentially necessary for the growth of *ARID1A*-mutated cancer cell lines in the project, ‘Achilles’ [28]. A dependency of *ARID1A*-mutated tumors on activation of the PI3K/AKT pathway is further supported by a study in ARID1A-knockout and ARID1A/PTEN-double-knockout mice, where it was observed that only mice with simultaneous loss of ARID1A and PTEN expression developed poorly differentiated ovarian tumors, in contrast to mice with loss of only ARID1A that did not develop ovarian tumors [29]. This indicates that ARID1A-mutated cell clones require a second-hit mutation in order to transform into cancer, which may explain why loss of ARID1A expression was observable in a subset of non-atypical benign endometriosis in one of our previous studies [30].

Interestingly, *ARID1A*-mutated OCCC have been reported to be associated with resistance to conventional platinum-based chemotherapy regimens in a recent study [31]. If this observation is confirmed in larger collectives, an analysis of the ARID1A expression status, which is easily feasible by immunohistochemistry, will possibly become an important predictive tool to assess the probability of resistance to conventional platinum based chemotherapy and, as we suggest in this study, increased sensitivity to PI3K- and/or AKT-inhibitors. PI3K/AKT-inhibitors, which are a promising tool to overcome chemotherapy resistance, may be selectively added to the treatment strategy in patients with ARID1A-deficient tumors.

In conclusion, ARID1A deficiency leads to a significantly increased sensitivity towards PI3K- and AKT-inhibition in tumor cells *in vitro*. The findings suggest a specific requirement of the PI3K/AKT pathway in ARID1A-deficient cancer cells and reveal a synthetic lethal interaction between loss of ARID1A expression and inhibition of the PI3K/AKT pathway.

## MATERIAL AND METHODS

### Cell culture

The breast carcinoma cell line MCF7 (E. Felley-Bosco, Switzerland) and the human primary lung fibroblasts MRC5 (ATCC) were grown in Dulbecco's modified Eagle's medium (DMEM) (Gibco, LifeTechnologies, Thermo Fisher Scientific Inc., Waltham, MA, USA). The OCCC cell lines ES-2, RMG-1, OVSAYO, OVISE, HCH-1 (R. Natrajan, UK) were grown in RPMI 1640 medium (Gibco). All cell lines used in this study were verified by short tandem repeat (STR) DNA identity testing. All growing media were supplemented with 10% (v/v) fetal bovine serum (Gibco) and Antibiotic-Antimycotic (containing penicillin, streptomycin and amphotericin B) (Gibco). Cell cultures were maintained at 37°C in a humidified atmosphere at 5% CO2. Cells were passaged at 90% confluency.

### Transient siRNA transfections

Transient knockdown of ARID1A was performed with four different ARID1A siRNAs (J-017263-05, -06, -07, and -08, ON-TARGETplus Human ARID1A siRNA, Dharmacon, Thermo Scientific, Thermo Fisher Scientific Inc., Waltham, MA, USA) using RNAimax (Gibco) according to the manufacturer's instructions. The control cells were transfected with siRNA against luciferase (sense): CGUACGCGGAAUACUUCGATTdTd (Microsynth AG, Baldach, Switzerland). The AKT1 knockdown control experiments were performed with a siRNA smart-pool (L-003000-00, ON-TARGET plus Human AKT1 (207) siRNA, Dharmacon). Cells were transfected overnight and the medium was replaced the following morning. The size of culture dishes and length of transfection is indicated for the specific experiments.

### Treatment with AKT- and PI3K- inhibitors

The AKT-inhibitors MK-2206, perifosine, and the pan-PI3K-inhibitor buparlisib (BKM120) were all obtained from Selleckchem.com (Selleck Chemicals, Houston, TX, USA). The inhibitors were diluted according to the manufacturer's protocol (MK-2206 and buparlisib in DMSO and perifosine in sterile ddH_2_O) and prepared freshly for each treatment. Vehicle substance was used in the highest corresponding concentration for the treatment of the controls.

### Drug sensitivity assays

Drug sensitivity assays were performed according to the drug manufacturer's recommendations (Selleckchem.com). In brief, cells were seeded in 96-well plates at concentrations of 1000-2000 cells per well (MRC5 cells were seeded at a density of 10'000 cells per well). On the following day, cells were transiently transfected overnight with the corresponding siRNAs and then treated with the corresponding drugs at the indicated concentrations or vehicle substance as described above. Treatment was repeated after 2-3 days. After a total period of 4-5 days' treatment, the medium was replaced by phenol-red free medium and the quantity of viable cells was assessed using a MTS assay (Cell Titer Aqueous, Promega, Madison, WI, USA) according to the manufacturer's protocol. Plate absorbances were obtained using a spectrophotometer (Epoch Microplate Spectrophotometer, BioTek, Winooski, VT, USA). Each experiment was performed on at least two separate occasions for every unique ARID1A siRNA sequence and each of them run in triplicates.

### Protein extraction and immunoblotting

Cells were seeded in 6cm culture dishes and transfected with the corresponding siRNAs the following day as described above, followed by the drug treatment where indicated. Whole-cell protein extracts were prepared from cells lysed before reaching confluence with an SDS lysis buffer. Western blotting was performed with primary antibodies against ARID1A (ab50878, Abcam, Cambridge, UK), AKT1 (#9272, Cell Signaling Technology, Danvers, MA, USA), phopho-Ser473-AKT (pAKT-Ser^473^) (#4058, Cell Signaling Technology), phospho-Thr421/Ser424-p70 S6 Kinase (pS6K) (#9204, Cell Signaling Technology,), PARP-1 (#9542, Cell Signaling Technology), beta-Actin (A5441, Sigma-Aldrich, Buchs SG, Switzerland). Incubation with the primary antibody was followed by incubation with a HRP-conjugated secondary antibody (anti-mouse and anti-rabbit HRP, Sigma-Aldrich) and chemiluminescent detection of proteins (Amersham, GE Healthcare Bio-Sciences, Pittsburgh, PA, USA).

### TUNEL assay and fluorescence microscopy

A TUNEL assay (In Situ Cell Death Detection Kit, Fluorescein, Roche, Basel, Switzerland) based on labelling of DNA strand breaks was used to directly visualize apoptosis in the treated cell cultures. In brief, cells were directly plated on microscopy coverslips transfected with siRNAs and treated for 60h with MK-2206 or vehicle substance as described above. Positive controls were obtained by treatment of fixated non-apoptotic cells with 30U/l recombinant DNase (in 1% (w/v) BSA / PBS) for 1h immediately prior to the addition of the incubation mix. Negative controls were obtained by omission of the TUNEL enzyme in the incubation mix. The coverslips were mounted in DAPI mounting medium (Vectashield, Reactolab SA, Servion, Switzerland). A Leica DMI6000B microscope was used for analysis of the specimens.

### Statistical analysis

Statistical analysis was performed with graph pad prism version 6 (GraphPad Software Inc., La Jolla, CA, USA). Results are expressed as mean ± standard error of the mean (SEM). Significance was assumed if p<0.05 (F-test) in comparison to the respective controls.
